# Construction and Evaluation of a Risk Prediction Model for Stress Urinary Incontinence in Late Pregnancy Based on Clinical Factors and Pelvic Floor Ultrasound Parameters

**DOI:** 10.3390/diagnostics15131630

**Published:** 2025-06-26

**Authors:** Shunlan Liu, Aizhi Huang, Yubing Huang, Linlin Hu, Lihong Cai, Shaozheng He, Guorong Lyu, Xihua Lian

**Affiliations:** 1 Department of Ultrasound Medicine, Second Affiliated Hospital of Fujian Medical University, Quanzhou 362000, China; 85443785@fjmu.edu.com (S.L.); z994069607@sina.com (Y.H.); hsz@fjmu.edu.cn (S.H.); 2 Department of Ultrasound Medicine, Nanan Hospital, Quanzhou 362300, China; huangaizhi123@sina.com; 3 Department of Obstetrics and Gynecology, Second Affiliated Hospital of Fujian Medical University, Quanzhou 362000, China; hll13655038672@163.com (L.H.); carolclh@sina.com (L.C.)

**Keywords:** stress urinary incontinence, late pregnancy, pelvic floor ultrasound, risk prediction, nomogram

## Abstract

**Background**: Stress urinary incontinence (SUI) is frequently underrecognized in late pregnancy, with limited tools for effective risk assessment. This study aimed to evaluate the predictive value of clinical and pelvic floor ultrasound parameters for SUI and construct a validated risk model. **Methods**: Clinical, obstetric, and pelvic floor ultrasound findings were collected from a total of 521 women in late pregnancy who were enrolled in the study. Based on follow-up results, participants were categorized into SUI and non-SUI groups. Logistic regression analyses were used to identify independent risk factors for SUI, which were incorporated into a nomogram. **Results**: Four independent predictors were identified: vaginal delivery history (odds ratio [OR] = 2.320), bladder neck funneling (OR = 2.349), bladder neck descent (OR = 1.891), and pubococcygeus muscle contraction strain rate (OR < 0.001). The nomogram achieved an AUC of 0.817 (95% CI: 0.770–0.863) in the training set and 0.761 (95% CI: 0.677–0.845) in the test set. **Conclusions**: The nomogram based on clinical and pelvic floor ultrasound parameters accurately predicts the risk of SUI during late pregnancy, offering a useful tool for early identification and personalized management.

## 1. Introduction

Stress urinary incontinence (SUI) refers to the involuntary leakage of urine during increased abdominal pressure. It is a common characteristic of pelvic floor dysfunction during pregnancy, with an incidence rate ranging from 38.7% to 51.5% [1]. SUI not only negatively affects daily life and sexual activity but also increases the risk of postpartum urinary incontinence [1,2,3]. Studies show that women who experience SUI symptoms during pregnancy are more than twice as likely to develop recurrent urinary incontinence within 15 years postpartum compared to asymptomatic pregnant women [4]. Pelvic floor muscle training (PFMT) is an effective intervention for preventing and controlling the occurrence of SUI during pregnancy. However, providing PFMT to all pregnant women would increase unnecessary medical and nursing burdens [5,6,7]. Therefore, early identification of high-risk pregnant women and implementation of interventions during pregnancy can help reduce the incidence of SUI during and after pregnancy while optimizing medical resource utilization [8].

Recent studies have explored predictive tools for SUI. With the growing depth of research, an increasing number of physiological factors are being identified as closely related to the occurrence of SUI during pregnancy. Studies indicate that uterine enlargement, increased abdominal pressure, and changes in the pelvic floor muscles are the primary physiological mechanisms for SUI development. Additionally, a history of urinary incontinence, instrumental delivery, and alcohol consumption are considered potential risk factors [9]. Other factors, such as maternal age (>35 years), multiple gestations, prolonged second-stage labor, history of delivering a macrosomic infant, increased body mass index (BMI) during pregnancy, and constipation, are also believed to contribute to the risk of SUI [10,11]. However, most existing studies mainly rely on questionnaire surveys, focusing on subjective symptoms and neglecting objective medical indicators, limiting the comprehensiveness and objectivity of risk assessment.

Transperineally Pelvic Floor Ultrasound (TPUS) is a non-invasive, radiation-free, and cost-effective examination method that allows real-time dynamic observation of changes in pelvic floor support structures and effective assessment of their function [12]. Studies have shown that pelvic floor ultrasound parameters, such as bladder neck rotation angle, bladder neck descent, bladder–urethra posterior angle, bladder protrusion, and bladder neck funneling, are closely related to SUI [12,13]. However, existing studies largely focus on the predictive or diagnostic value of individual ultrasound parameters for SUI, with most predicting postpartum SUI using prenatal pelvic floor ultrasound. Research on predicting SUI during pregnancy remains limited.

This study aimed to combine clinical factors and multiple ultrasound parameters to construct a comprehensive prediction model for SUI during late pregnancy. Through multivariate regression analysis, the independent and combined effects of various factors on SUI occurrence were assessed and used to develop a nomogram-based prediction model. Our study sought to provide scientific evidence for the accurate identification of high-risk pregnant women with late-pregnancy SUI and offer guidance for the rational use of PFMT.

## 2. Materials and Methods

### 2.1. Study Population

This was a prospective study conducted in accordance with the Declaration of Helsinki and approved by the Ethics Committee of the Second Affiliated Hospital of Fujian Medical University (Approval No. 2021Y160). Written informed consent was obtained from all participants. From May 2022 to November 2024, pregnant women in the third trimester (≥28 weeks gestation) who underwent Obstetric Ultrasound and TPUS examinations were recruited.

The inclusion criteria were as follows: (1) singleton pregnancy; (2) no urinary tract infections; and (3) the ability to effectively perform pelvic floor muscle contraction and the Valsalva maneuver [14,15]. The exclusion criteria included: (1) any symptoms of urinary incontinence prior to TPUS, including those occurring before pregnancy or during the first or second trimesters; (2) severe maternal-fetal complications such as preeclampsia, placenta previa, or low-lying placenta; and (3) refusal to participate or incomplete clinical data.

Sample Size Estimation: Based on the conventional rule-of-thumb for multivariate logistic regression, the minimum sample size for the less frequent category of the outcome variable should be at least 10 times the number of predictors [16]. In this study, the dependent variable had two levels: stress urinary incontinence (SUI) and non-stress urinary incontinence (non-SUI). With an estimated 10 meaningful predictors, the required sample size for the SUI group was calculated as 10 × 10 = 100 cases. Referring to literature data, the prevalence of pregnancy-related SUI ranges from 38.7% to 51.5% [1]. Assuming an incidence rate of 45%, the minimum total sample size needed for modeling was estimated to be 100/0.45 ≈ 222 participants.

**Figure 1:**
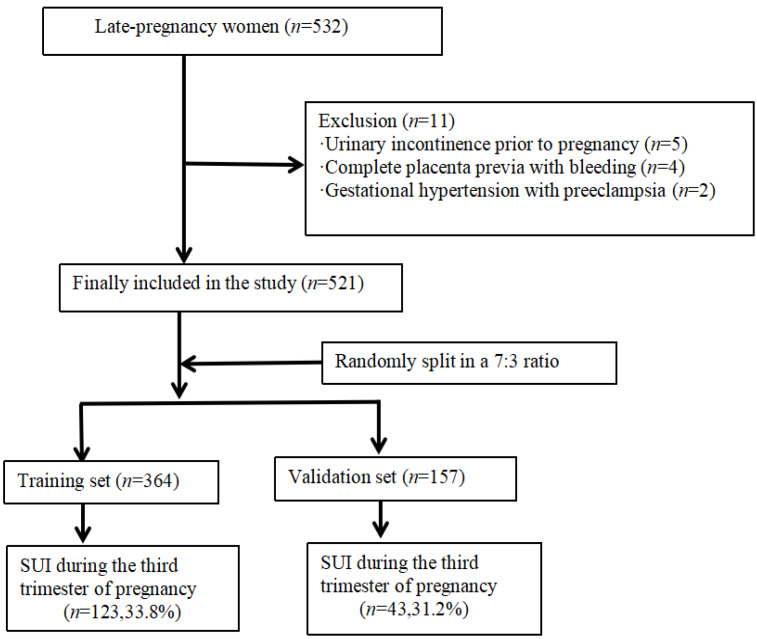
Flowchart of the inclusion of the study participants.

A total of 532 pregnant women were initially recruited. Among them, 5 women were excluded due to a pre-pregnancy diagnosis of urinary incontinence, 4 were diagnosed with complete placenta previa accompanied by bleeding, and 2 were diagnosed with hypertensive disorders of pregnancy with preeclampsia. Ultimately, 521 eligible pregnant women were included in the study. The dataset was randomly divided into a training set and a validation set at a 7:3 ratio using the sample function in R version 4.2.0 (R Foundation for Statistical Computing, Vienna, Austria). The training set was used for model development, while the validation set was used to independently evaluate model performance and generalizability. The flowchart of the inclusion of the study participants is in Figure 1.

### 2.2. Research and Examination Methods

#### 2.2.1. Obstetric Ultrasound

Obstetric ultrasound was performed using a GE Voluson E10 ultrasound diagnostic system (GE Healthcare, Zipf, Austria) equipped with a 4C convex array transducer, with a frequency range of 3–5 MHz. Fetal-related information including biparietal diameter (BPD), head circumference (HC), and fetal weight (FW) were assessed.

#### 2.2.2. Transperineally Pelvic Floor Ultrasound

**Figure 2:**
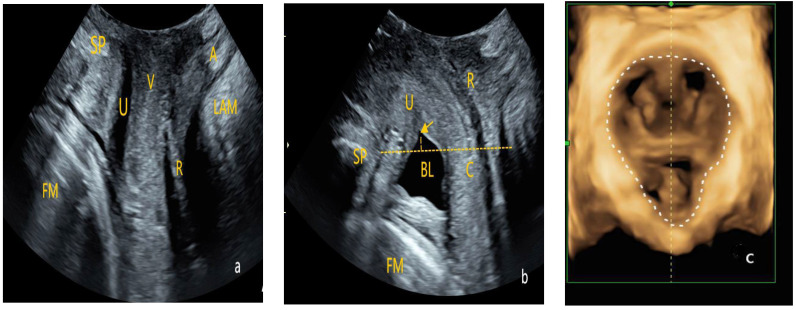
Transperineally pelvic floor ultrasound examination. (**a**) Two-dimensional pelvic floor ultrasonographic image in the mid-sagittal plane obtained at rest (FM: fetal head; SP: pubic symphysis; U: urethra; V: vagina; A: anal canal; R: rectal ampulla; LAM: levator ani muscle). (**b**) Two-dimensional pelvic floor ultrasonographic image during maximum Valsalva maneuver to assess bladder protrusion and urethral funneling (BL: bladder; Arrow: proximal urethra showing funneling; Yellow dashed line: a horizontal line drawn through the posterior-inferior border of the pubic symphysis). (**c**) Four-dimensional sonographic image of the levator ani muscle hiatus (White dashed line: the boundary of the hiatal area, which is the area enclosed by the pubic rami, the pubic symphysis, and the medial border of the puborectal muscle).

- Instrument: A GE Voluson E10 color Doppler ultrasound diagnostic system (GE Healthcare, Zipf, Austria) with an RAB4-8L volumetric probe, with a frequency range of 4–8 MHz, was used.
- Examination Method: After the patient emptied their bladder and bowels, they were positioned supine on the examination table with their legs flexed and mildly abducted in the lithotomy position. The specialized probe was wrapped and a large amount of disinfectant gel was applied to avoid air interference within the probe cover, ensuring good image quality. The probe was placed between the labia, and images of the pelvic floor in the mid-sagittal plane were obtained in three conditions—at rest, during maximal voluntary contraction, and during a Valsalva maneuver. The structures examined included the pubic symphysis, urethra, bladder, vagina, rectum, anal canal, and anorectal angle. Using the pubic symphysis as the central reference point, the sagittal plane images of the vagina, urethra, and anal canal were captured. Volume scanning was then performed, with appropriate adjustment of the selected region to collect two-dimensional and four-dimensional ultrasonographic images of the pelvic floor at rest, during maximal voluntary contraction, and during the Valsalva maneuver (Figure 2).

- Observation Indicators: (1) bladder neck vertical position, which is the vertical distance from the bladder neck to horizontal level of the posterior-inferior margin of the pubic symphysis; (2) bladder neck descent, which is the difference in the bladder neck position between the maximum Valsalva and resting states; (3) bladder–urethra posterior angle, which is the angle between the posterior wall of the bladder (trigonal region) and proximal urethra; (4) urethral rotation angle, which is the difference in the urethral inclination angle between the maximum Valsalva maneuver and resting states; (5) bladder neck funneling; (6) hiatal area (HA),the area enclosed by the pubic rami, pubic symphysis, and the medial border of the puborectal muscle; and (7) the levator ani muscle contraction strain rate, which is the difference in the hiatal area (HA) between the resting and contraction states, divided by the HA in the resting state.

The obstetric ultrasound and TPUS examinations were performed by two senior sonographers, each with over 5 years of experience in obstetric and pelvic floor ultrasonography, and both blinded to the participants’ clinical information. To evaluate intra-observer and inter-observer reliability, a random sample of 30 pregnant women was selected for reproducibility analysis. The primary sonographer (S.L.) conducted two separate measurements of all pelvic floor ultrasound parameters to assess intra-observer consistency. To evaluate inter-observer reliability, a second sonographer (X.L.) independently performed a separate set of measurements. Both sonographers conducted their assessments independently and were blinded to each other’s results to ensure objectivity.

#### 2.2.3. Clinical Data Collection

At the time of the initial pelvic floor ultrasound examination, clinical information was collected through participants interviews and an electronic medical record review by a trained research assistant who was independent of the study team. The collected data included demographic characteristics (such as age and body mass index [BMI]), obstetric history, history of miscarriage, previous delivery history, family history of pelvic floor disorders, smoking history, history of chronic cough, and history of medication use (including but not limited to diuretics, sedatives, and muscle relaxants).

After the baseline pelvic floor ultrasound, all participants were followed up weekly via telephone interviews until delivery. These follow-ups were primarily conducted to assess the occurrence of stress urinary incontinence (SUI) during the third trimester. The diagnosis of SUI was based on participants’ self-reported symptoms and was evaluated using the International Consultation on Incontinence Questionnaire-Short Form (ICIQ-SF) [17], a validated and widely used instrument with high sensitivity, specificity, and reliability for the assessment of urinary.

### 2.3. Statistical Methods

Data organization and analysis were performed using SPSS 26.0 statistical software (IBM Corp, Armonk, NY, USA). For normally distributed continuous data, the mean ± standard deviation ($\bar{x}\pm s$) was used, the groups were compared using the independent two-sample *t*-test. For data that did not follow a normal distribution, the median (interquartile range) was used, and the groups were compared using the Mann–Whitney *U* test. Categorical data were expressed as frequency (percentage), and group comparisons were performed using the chi-square test. An intraclass correlation coefficient (ICC) or Kappa was used to analyze the consistency of intra- and inter-operators. Multivariate analysis was conducted using binary logistic regression, with the “Forward LR” method for variable selection. Specifically, the “Forward LR” method begins with an empty model and progressively adds variables that are most significantly associated with the outcome. Each variable is assessed based on its *p*-value, with a threshold of 0.05 for inclusion in the model. A nomogram was constructed based on this model using R 4.2.0 statistical software(R Foundation for Statistical Computing, Vienna, Austria). The predictive performance of the model was assessed using receiver operating characteristic (ROC) curves, and the DeLong test was employed to assess whether there were statistically significant differences between the areas under the curve (AUC). A *p*-value < 0.05 was considered statistically significant. The model was validated using 1000 bootstrap resamples, and the Hosmer–Lemeshow test was used to assess the goodness-of-fit of the model. A *p*-value > 0.05 indicated a good model fit, and calibration plots were used to test the model’s accuracy. Decision curve analysis plots were drawn using R 4.2.0 software to evaluate the clinical utility of the model.

## 3. Results

### 3.1. Consistency Analysis

The consistency for intra- and inter-operator measurements were good, with the intraclass correlation coefficient (ICC) or Kappa value exceeding 0.75 for bladder neck mobility, rotation angle, the bladder–urethra posterior angle, bladder neck funneling, the bladder lowest point and levator ani muscle contraction strain rate (Table 1).

### 3.2. Comparison of the Demographic Data Between the Training and Validation Sets

The data set was randomly split into the training and validation sets in a 7:3 ratio. A comparison of the demographic data between the training and validation sets, including age, BMI, gestational week, number of pregnancies, number of vaginal deliveries, number of cesarean sections, number of miscarriages, number of late induced abortions, family history of pelvic floor disorders, smoking history, history of chronic cough, and history of medication use, biparietal diameter, head circumference, fetal weight, bladder neck mobility, rotation angle, bladder–urethra posterior angle, bladder lowest point, levator ani muscle contraction strain rate, bladder neck funneling, and incidence of urinary incontinence, showed no statistically significant differences (*p* > 0.05), as shown in Table 2.

### 3.3. Univariate Analysis of Factors Affecting the Occurrence of SUI Based on the Training Set

A comparison of BMI, gestational week, number of cesarean sections, number of miscarriages, number of late induced abortions, family history of pelvic floor disorders, smoking history, history of chronic cough, and history of medication use, biparietal diameter, head circumference, fetal weight, and rotation angle between pregnant women with and without SUI revealed no statistically significant differences (*p* > 0.05). However, women with SUI had a higher age, number of pregnancies, number of vaginal deliveries, bladder neck mobility, bladder–urethra posterior angle, and bladder neck funneling rate compared to those without SUI (*p* < 0.05). In contrast, women with SUI had a lower bladder lowest point and levator ani muscle contraction strain rate compared to those without SUI (*p* < 0.05). These findings are shown in Table 3.

### 3.4. Multivariate Logistic Regression Analysis of Factors Affecting the Occurrence of SUI Based on the Training Set

SUI occurrence was set as the dependent variable (assigned value 1 for “yes” and 0 for “no”). Variables that showed differences in the univariate analysis of factors affecting SUI were included in the multivariate model. Since bladder neck mobility represents the difference in the bladder lowest position between the two states and correlates with the bladder lowest point, bladder neck mobility was selectively included as an independent variable to reduce collinearity. The final model included bladder neck funneling, age, number of pregnancies, number of vaginal deliveries, bladder neck mobility, levator ani muscle contraction strain rate, and bladder–urethra posterior angle as independent variables for binary logistic regression analysis.

**Figure 3:**
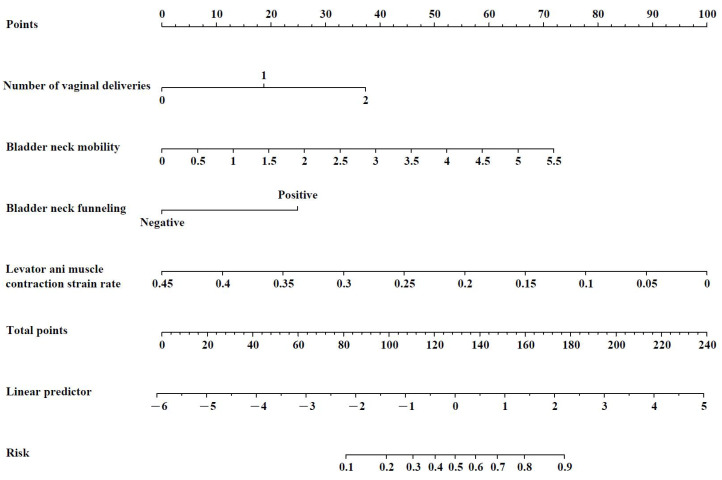
Multivariable regression nomogram for factors influencing the occurrence of stress urinary incontinence.

The results show that bladder neck funneling, a higher number of vaginal deliveries, greater bladder neck mobility, and lower levator ani muscle contraction strain rate were independent risk factors for SUI. Based on the binary logistic regression analysis, the regression equation for the model was as follows: Logit(*p*) = −1.326 + 0.841 × number of vaginal deliveries + 0.637 × bladder neck mobility + 0.854 × bladder neck funneling −10.128 × levator ani muscle contraction strain rate, as shown in Table 4. The nomogram derived from the multivariate regression of factors affecting the occurrence of SUI is presented in Figure 3.

### 3.5. Consistency Test of Predictive Performance Between the Training and Validation Set Models

**Figure 4:**
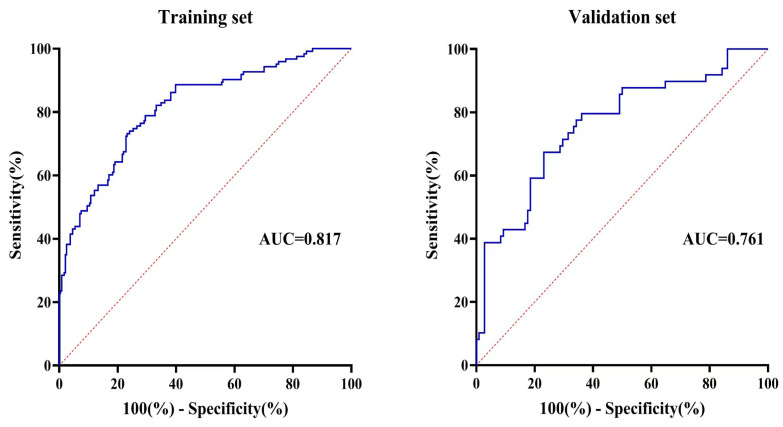
Receiver operating characteristic (ROC) curve for predictive performance of the training and validation sets. The blue solid lines correspond to the ROC curves of the training and validation sets, respectively, while the red dashed line denotes the reference line (AUC = 0.5), indicating no discriminative ability.

SUI occurrence was set as the status variable (assigned value 1 for “yes” and 0 for “no”). Based on the logistic regression equation model described above, a combined predictive model was developed for the training and validation sets, and an ROC curve analysis was performed. The results show that the area under the curve (AUC) for the training set model predicting SUI occurrence was 0.817 (95% confidence interval [CI]: 0.770–0.863) (*p* < 0.001), with a specificity of 0.768, sensitivity of 0.732, Youden index of 0.500, positive predictive value of 0.616, and negative predictive value of 0.849. For the validation set model, the AUC for predicting SUI occurrence was 0.761 (95% CI: 0.677–0.845) (*p* < 0.001), with a specificity of 0.769, sensitivity of 0.673, Youden index of 0.442, positive predictive value of 0.569, and negative predictive value of 0.838. A comparison of the AUCs between the training and validation set models for predicting SUI occurrence revealed no statistically significant difference (*p* = 0.2574). These results are presented in Table 5 and Figure 4.

Additionally, the AUCs for individual predictors of SUI occurrence in the training set model were as follows: bladder neck mobility, 0.722 (95% CI: 0.666–0.778, *p* < 0.001); levator ani muscle contraction strain rate, 0.706 (95% CI: 0.653–0.760, *p* < 0.001); bladder neck funneling, 0.651 (95% CI: 0.589–0.713, *p* < 0.001); and number of vaginal deliveries, 0.644 (95% CI: 0.581–0.707, *p* < 0.001).

### 3.6. Consistency Test Between the Training and Validation Set Models

**Figure 5:**
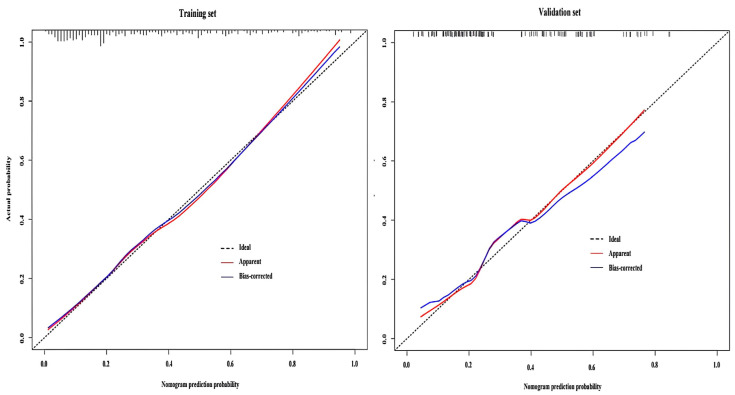
Calibration curve of the stress urinary incontinence model for the training and validation sets.

Bootstrap resampling was performed 1000 times for model validation. The Hosmer–Lemeshow test revealed no statistically significant difference between the actual and predicted probabilities of SUI occurrence in the training set (χ^2^ = 13.767, df = 8, *p* = 0.088) or validation set (χ^2^ = 9.376, df = 8, *p* = 0.312). Calibration plots for the training and validation set models showed a high level of consistency between model predictions and actual clinical observations (Figure 5).

### 3.7. Clinical Utility Test of the Training and Validation Set Models

**Figure 6:**
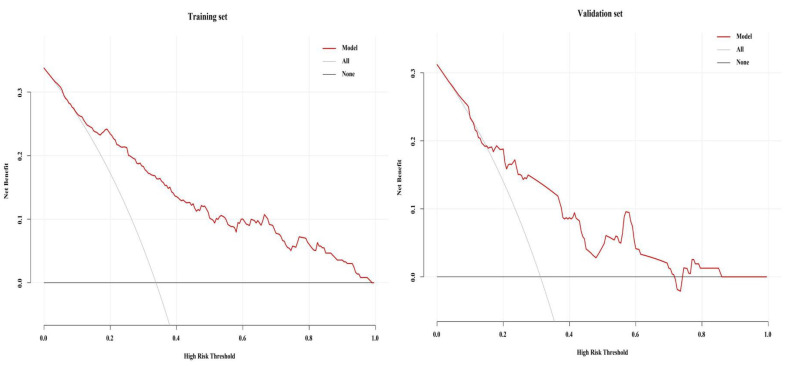
Decision curve of the stress urinary incontinence model for the training and validation sets.

As shown in Figure 6, the training set model predicted the probability of SUI occurrence (i.e., threshold probability) within the range of 11% to 100%. The validation set model predicted the probability of SUI occurrence (i.e., threshold probability) within the ranges of 16% to 72% and 76% to 85%. In both models, the prediction ranges were above the two extreme lines (i.e., the “All” line assuming all patients have SUI and receive treatment and the “None” line assuming none of the have SUI and do not receive treatment). This indicates that using the two models to predict SUI and guide treatment decisions can provide clinical benefits, demonstrating good clinical utility.

**Figure 7:**
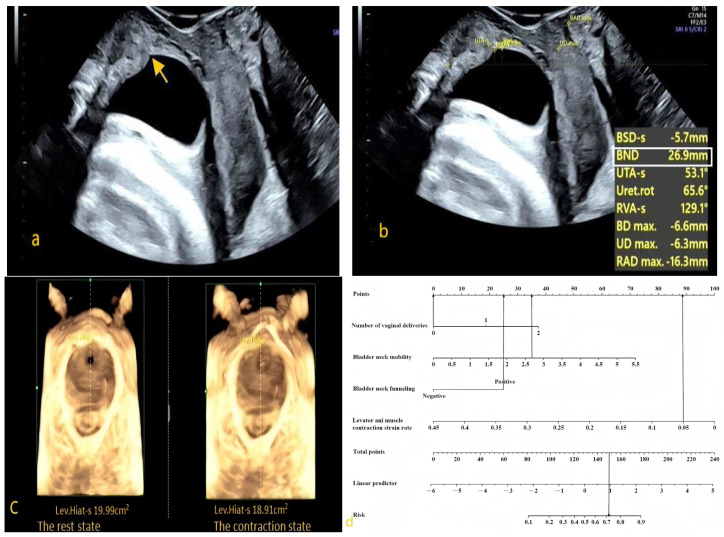
Example of the application of the nomogram. (**a**) Bladder neck funneling under the Valsalva maneuver (yellow arrow); (**b**) Bladder neck mobility (white rectangular box); (**c**) Puborectal muscle hiatus area under the resting and pelvic floor muscle contraction states; (**d**) Pelvic floor ultrasound examination showing bladder neck funneling (25 points), bladder neck mobility of 2.69 cm (35 points), and puborectal muscle contraction strain rate of 0.05 (89 points). The total score is approximately 149 points (combining 25 + 0 + 35 + 89), predicting a 71.2% probability of stress urinary incontinence for this patient.

When using the nomogram, the score corresponding to each predictor is summed to obtain the total score, which can then be used to predict the probability of SUI occurrence. An example of the nomogram application is illustrated in Figure 7, showcasing the case of a pregnant woman with no previous vaginal delivery (0 point) who underwent pelvic floor ultrasound at 32 weeks of pregnancy. The results showed bladder neck funneling (25 points), bladder neck mobility of 2.69 cm (35 points), and a levator ani muscle contraction strain rate of 0.05 (89 points). The total score sums up to 149 points (combining 25 + 0 + 35 + 89). Therefore, the predicted probability of this pregnant woman developing SUI during pregnancy was approximately 71.2%. Follow-up at 36 weeks of pregnancy confirmed the occurrence of SUI symptoms.

## 4. Discussion

Pregnancy-related SUI is a common condition that significantly impacts the quality of life of pregnant women and may increase the risk of postpartum SUI. Therefore, timely identification and effective interventions are crucial to mitigate its negative effects. Currently, risk prediction of pregnancy-related SUI [18,19] primarily relies on clinical data (such as BMI, obstetric history, and pregnancy symptoms); however, an objective risk assessment system is still lacking. Based on the theory of the pelvic floor anatomy, the occurrence of SUI is closely related to damage to the pelvic organ support structures and connective tissue ligaments [20]. Imaging examinations, particularly pelvic floor ultrasound, provide reliable means for assessing changes in pelvic floor anatomy and have significant advantages in predicting SUI [21,22]. In this study, we analyzed the relationship between late-pregnancy SUI, clinical risk factors, and pelvic floor function and developed a comprehensive predictive model.

Our findings reveal four independent risk factors for pregnancy-related SUI, including bladder neck funneling, the number of vaginal deliveries, bladder neck mobility, and the levator ani muscle contraction strain rate. Specifically, an increased number of vaginal deliveries, greater bladder neck mobility, and a lower strain rate of levator ani muscle contraction were all associated with a higher risk of developing stress urinary incontinence. Previous studies [10,19] have demonstrated a close relationship between vaginal delivery and pregnancy-related SUI, corroborating our study’s findings. Additionally, increased bladder neck mobility (>2.4 cm) and bladder neck funneling have also been confirmed as risk factors for SUI, and the underlying mechanism may be related to the weakening of pelvic floor support structures, leading to increased bladder neck mobility [23]. These findings are consistent with those of previous studies [12,24] and further confirm the important role of changes in pelvic floor anatomy in the occurrence of SUI.

The levator ani muscle contraction strain rate reflects the ratio of the change in the HA of the levator ani muscle between the resting and contraction states. Thyer et al. [25] were the first to introduce the concept of strain rate in the ultrasonographic assessment of postpartum pelvic floor muscle function, finding a statistically significant correlation between the strain rate and pelvic floor muscle strength. In this study, the levator ani muscle contraction strain rate was introduced as a novel predictor of late-pregnancy SUI. The contraction ability of the levator ani muscle is crucial for maintaining normal urinary control. During pregnancy, the pelvic floor muscles may undergo subtle damage, and if these injuries are not adequately compensated, they may lead to permanent functional impairment, which can contribute to the development of SUI and other pelvic floor dysfunctions. In this study, transperineal pelvic floor ultrasound was used to accurately quantify the strain rate of the levator ani muscle, reducing the influence of individual differences. The findings revealed that the levator ani muscle contraction strain rate was significantly lower in the SUI group than in the non-SUI group, indicating that impaired contraction function of the levator ani muscle may be a key contributor to the development of SUI.

The main contribution of this study is the development of a multivariable prediction model for assessing the risk of late pregnancy-related SUI, incorporating clinical factors and pelvic floor ultrasound parameters. Compared to univariate models, the integrated model demonstrates stronger overall predictive ability. Additionally, the AUC value of the integrated model in this study was 0.817, which is higher than the model developed by Liang et al. [19]. Based on pregnancy-related BMI, constipation, and previous delivery history (AUC = 0.787), these results suggest that the combination of clinical factors and imaging data significantly enhances the accuracy of SUI prediction, in line with the findings of Chen et al. [26]. Although the AUC was lower in the validation set than in the training set, the difference was not statistically significant, indicating consistent predictive performance across datasets. Minor discrepancies in AUC between training and validation sets are commonly observed in predictive modeling and may be attributed to slight overfitting or random variability between datasets [27,28]. Importantly, the absence of a significant difference in AUC supports the generalizability of the model. Nevertheless, external validation in larger and more diverse populations is warranted to further confirm the model’s robustness and its applicability across different clinical settings.

To further validate the clinical utility of the model, we performed decision curve analysis. The results demonstrate that, across different threshold ranges, the predictive probabilities of both the training and validation set outperformed extreme assumptions (i.e., assuming all patients are either SUI-positive or SUI-negative). This indicates that applying this model to predict the risk of SUI in pregnant women can significantly improve clinical decision-making outcomes and help avoid unnecessary treatments or overlooking high-risk patients. Moreover, the developed nomogram provides clinicians with a simple tool to quickly calculate the risk of SUI by summing the scores of individual predictive factors. This information can assist clinicians in accurately identifying high-risk pregnant women for late pregnancy-related SUI and provide a scientific basis for the appropriate allocation of pelvic floor muscle training (PFMT) resources.

Nevertheless, several limitations of this study should be acknowledged. First, the data were collected from a single medical institution, which may limit the generalizability of the findings. Second, although various physiological and clinical factors were included, other potentially relevant variables—such as a history of heavy lifting, alcohol consumption, fluid intake habits, and physical activity levels—were not collected, which may have affected the comprehensiveness of the predictive model. Future studies should aim to validate the predictive model in multicenter cohorts to improve its external applicability. In addition, further exploration of additional potential influencing factors is warranted to enhance the model’s accuracy and robustness.

## 5. Conclusions

This study introduced a multivariable risk prediction model for pregnancy-related SUI, incorporating clinical factors and pelvic floor ultrasound parameters. The independent risk factors for SUI identified were bladder neck mobility, the puborectal muscle contraction strain rate, number of vaginal deliveries, and bladder neck funneling. The model demonstrated good predictive ability and provides a valuable tool for early detection and personalized management of SUI in the late stages of pregnancy.

## Figures and Tables

**Table 1:** Consistency analysis of intra-operator and inter-operator.

Parameters	Intra-Operator		Inter-Operator	
	ICC/Kappa	95% CI	ICC/Kappa	95% CI
Bladder neck mobility	0.8947	0.7855~0.9498	0.8070	0.6250~0.9058
Rotation angle	0.9876	0.9734~0.9943	0.9740	0.9447~0.9879
Bladder–urethra posterior angle	0.9122	0.8195~0.9584	0.8910	0.7784~0.9480
Bladder neck funneling	0.8871	0.67118~1.0000	0.7879	0.50684~1.0000
Bladder lowest point	0.9911	0.9809~0.9959	0.9590	0.9135~0.9808
Levator ani muscle contraction strain rate	0.9815	0.9605~0.9914	0.8939	0.7841~0.9495

Note: ICC: intraclass correlation coefficients; 95% CI: 95% confidence interval.

**Table 2:** Comparison of demographic data between the training and validation sets.

Indicator	Total Number of Cases (*n* = 521)	Training Set (*n* = 364)	Validation Set (*n* = 157)	χ^2^/*t*/*z* Value	*p* Value
Age (years)	29.00 (26.00, 31.00)	28.50 (25.25, 31.00)	29.00 (26.00, 31.00)	−1.051 ^b^	0.293
BMI (kg/m^2^)	26.83 (24.17, 29.85)	26.78 (24.25, 30.00)	26.94 (23.90, 29.67)	−0.185 ^b^	0.854
Gestational age (weeks)	31.00 (29.00, 34.00)	31.00 (29.00, 34.00)	31.00 (30.00, 34.00)	−1.108 ^b^	0.929
Smoking history, *n* (%)				0.679 ^c^	0.410
NO	503 (96.5)	353 (97.0)	150 (95.5)		
YES	18 (3.5)	11 (3.0)	7 (4.5)		
History of chronic cough, *n* (%)				0.644 ^c^	0.422
NO	494 (94.8)	347 (95.3)	147 (93.6)		
YES	27 (5.2)	17 (4.7)	10 (6.4)		
Family history of pelvic floor disorders, *n* (%)				0.388 ^c^	0.534
NO	468 (89.8)	325 (89.3)	143 (91.1)		
YES	53 (10.2)	39 (10.7)	14 (8.9)		
History of medication use, *n* (%)				0.011 ^c^	0.916
NO	497 (95.4)	347(95.3)	150 (95.5)		
YES	24 (4.6)	17(4.7)	7 (4.5)		
Number of pregnancies	2.00 (1.00, 2.00)	2.00 (1.00, 2.00)	2.00 (1.00, 2.00)	−0.740 ^b^	0.459
Number of vaginal deliveries	(0, 1)	0 (0, 1)	0 (0, 0)	−1.232 ^b^	0.218
Number of cesarean sections	0 (0, 0)	0 (0, 0)	0 (0, 0)	−0.946 ^b^	0.344
Number of miscarriages	0 (0, 1)	0 (0, 1)	0 (0, 1)	−0.711 ^b^	0.477
Number of late induced abortion	0 (0, 0)	0 (0, 0)	0 (0, 0)	−0.939 ^b^	0.348
Biparietal diameter (cm)	8.00 (7.54, 8.63)	8.00 (7.50, 8.50)	8.20 (7.69, 8.85)	−0.291 ^b^	0.949
Head circumference (cm^2^)	28.90 (27.61, 30.75)	28.80 (27.53, 30.50)	29.61 (27.71, 31.05)	−1.927 ^b^	0.494
Fetal weight (kg)	2.00 (1.58, 2.56)	2.00 (1.55, 2.45)	2.18 (1.72, 2.76)	−0.291 ^b^	0.946
Bladder neck mobility (cm)	1.97 (1.33, 2.52)	1.98 (1.31, 2.57)	1.92 (1.41, 2.41)	−0.554 ^b^	0.580
Rotation angle (°)	35.10 (25.20, 50.60)	36.15 (25.30, 51.70)	33.00 (24.50, 47.32)	−1.409 ^b^	0.159
Bladder–urethra posterior angle (°)	139.64 ± 22.07	140.39 ± 21.59	137.89 ± 23.12	1.185 ^a^	0.237
Bladder lowest point (cm)	0.74 (0.25, 1.31)	0.72 (0.23, 1.23)	0.85 (0.27, 1.45)	−0.958 ^b^	0.338
Levator ani muscle contraction strain rate	0.12 (0.06, 0.18)	0.12 (0.06, 0.18)	0.11 (0.05, 0.17)	−1.331 ^b^	0.183
Bladder neck funneling, *n* (%)				3.442 ^c^	0.064
NO	371 (71.2)	268 (73.6)	103 (65.6)		
YES	150 (28.8)	96 (26.4)	54 (34.4)		
Incidence of urinary incontinence, *n* (%)				0.330 ^c^	0.565
NO	349 (67.0)	241 (66.2)	108 (68.8)		
YES	172 (33.0)	123 (33.8)	49 (31.2)		

Note: ^a^: indicates the use of an independent samples *t*-test; ^b^: indicates the use of the Mann–Whitney U test; and ^c^: indicates the use of the chi-square test.

**Table 3:** Univariate analysis of factors influencing the occurrence of stress urinary incontinence based on the training set.

Indicator	No Stress Urinary Incontinence (*n* = 241)	Stress Urinary Incontinence (*n* = 123)	χ^2^/t/z Value	*p* Value
Age (years)	28.00 (25.00, 31.00)	29.00 (26.00, 32.00)	−2.481 ^b^	0.013
BMI (kg/m^2^)	26.68 (24.45, 29.95)	26.81 (23.34, 30.04)	−0.757 ^b^	0.449
Gestational age (weeks)	31.00 (29.00, 34.00)	31.00 (30.00, 34.00)	0.079 ^b^	0.937
Smoking history, *n* (%)			#	1
NO	234 (97.1)	119 (96.7)		
YES	7 (2.9)	4 (3.3)		
History of chronic cough, *n* (%)			0.153 ^c^	0.696
NO	229 (95.0)	118 (95.9)		
YES	12 (5.0)	5 (4.1)		
Family history of pelvic floor disorders, *n* (%)			0.285 ^c^	0.610
NO	216 (89.6)	109 (88.6)		
YES	25 (10.4)	14 (11.4)		
History of medication use, *n* (%)			0.435 ^c^	0.510
NO	231 (95.9)	116 (94.3)		
YES	10 (4.1)	7 (5.7)		
Number of pregnancies	2.00 (1.00, 2.00)	2.00 (1.00, 3.00)	−3.254 ^b^	0.001
Number of vaginal deliveries	(0,0)	0 (0,1)	−5.756 ^b^	<0.001
Number of cesarean sections	0 (0,0)	0 (0,0)	−0.333 ^b^	0.739
Number of miscarriages	0 (0,1)	0 (0,1)	−0.951 ^b^	0.341
Number of late induced abortions	0 (0,0)	0 (0,0)	−0.291 ^b^	0.771
Biparietal diameter (cm)	7.90 (7.50, 8.50)	8.00 (7.50, 8.50)	0.144 ^b^	0.885
Head circumference (cm^2^)	28.40 (27.50, 30.69)	28.90 (27.60, 30.30)	−0.22 ^b^	0.826
Fetal weight (kg)	1.91 (1.55, 2.45)	2.00 (1.55, 2.45)	0.146 ^b^	0.884
Bladder neck mobility (cm)	1.77 (0.88, 2.27)	2.50 (1.89, 2.99)	−6.935 ^b^	<0.001
Rotation angle (°)	36.00 (25.70, 50.55)	38.50 (25.00, 54.72)	−0.895 ^b^	0.371
Bladder–urethra posterior angle (°)	137.16 ± 20.77	146.72 ± 21.85	−4.081 ^a^	<0.001
Bladder lowest point (cm)	0.80 (0.36,1.31)	0.57 (−0.21, 1.51)	−2.810 ^b^	0.005
Levator ani muscle contraction strain rate	0.15 (0.09,0.21)	0.08 (0.04, 0.14)	−6.443 ^b^	<0.001
Bladder neck funneling, *n* (%)			38.146 ^c^	<0.001
NO	202 (83.8)	66 (53.7)		
YES	39 (16.2)	57 (46.3)		

Note: ^a^: indicates the use of an independent samples *t*-test; ^b^: indicates the use of the Mann–Whitney U test; and ^c^: indicates the use of the chi-square test; #: Fisher’s exact test was used; no corresponding test statistic is available.

**Table 4:** Multivariate regression analysis of factors influencing the occurrence of stress urinary incontinence based on the training set.

Indicator	β	SE	Wald χ^2^	*p* Value	OR	95% CI	
						Lower	Upper
Number of vaginal deliveries	0.841	0.206	16.627	<0.001	2.320	1.548	3.476
Bladder neck mobility	0.637	0.161	15.578	<0.001	1.891	1.378	2.595
Bladder neck funneling	0.854	0.296	8.305	0.004	2.349	1.314	4.199
Levator ani muscle contraction strain rate	−10.128	1.949	27.002	<0.001	<0.001	<0.001	0.002
Constant	−1.326	0.400	11.006	0.001	0.266		

Note: SE: standard error; OR: odds ratio; CI: confidence interval.

**Table 5:** Risk prediction performance analysis for adverse prognosis.

Indicator	Cutoff Value	AUC (95% CI)	Specificity	Sensitivity	Youden Index	Positive Predictive Value	Negative Predictive Value
Training set	0.372	0.817 (0.770–0.863)	0.768	0.732	0.500	0.616	0.849
Validation set	0.320	0.761 (0.677–0.845)	0.769	0.673	0.442	0.569	0.838

Note: AUC, area under the curve; CI, confidence interval.

## Data Availability

Due to ethical and privacy considerations, the datasets generated and/or analyzed during the current study are not publicly available. However, all data have been de-identified prior to sharing, with personal identifiers removed or coded. De-identified data may be made available from the corresponding author upon reasonable request, subject to appropriate ethical approval, with access strictly controlled to ensure participant privacy protection. This approach ensures necessary scientific transparency while safeguarding privacy.

## Abbreviations

The following abbreviations are used in this manuscript:

SUI	stress urinary incontinence
PFMT	pelvic floor muscle training
BMI	body mass index
AUC	area under the curve
HA	hiatal area
ROC	receiver operating characteristic
OR	odds ratio
CI	confidence interval
